# Diseases of the Heart and Circulation

**Published:** 1903-01-10

**Authors:** 


*(To be continued.)*

### DISEASES OF THE HEART AND CIRCULATION.

**The Diagnosis of Heart Disease** is the subject of a practical paper by Drummond.[1] Functional bruits are common in highly nervous people, especially in cases of chronic palpitation. They are usually systolic, and are best heard a little to the inner side of the apex beat, or in the neighbourhood of the tricuspid valves. Some are loudest when the patient is standing and disappear when he is at rest. They commonly disappear when the stethescope is firmly pressed against the chest wall. Want of knowledge of their existence often leads to mistakes in diagnosis, especially in connection with life assurance. It is not well known that permanent mitral regurgitation is often caused by alcohol. In the acute variety general œdema is an early symptom, and often affects face, body, and limbs in such a way as to suggest acute Bright's disease: the pulse is small, rapid, and usually compressible, whilst there is a striking disparity between the pulse and heart beat as seen and felt. In such cases digitalis is of little service, whilst strychnine often does good: but the prognosis is always grave, and the patient is liable to sudden death. Syphilis is a frequent cause of aortic disease. The patients, usually men, generally complain of pain in the chest, whilst the face presents the appearance of health. Pulsation in the episternal notch, associated with double aortic murmur and pain, in early middle life generally indicates a syphilitic lesion. It is in syphilitic cases that a history of ruptured valves is to be met, as it is common for the symptoms to begin suddenly. There are few more valuable physical sounds than the reduplicated first sound of kidney disease, best heard between the mitral and tricuspid areas, for it not only suggests the renal origin of the cardiac disturbance but indicates that compensation has begun to fail. In addition, as is well known, the aortic second sound is frequently accentuated. The cases which present the greatest difficulty are those where there is no albumen and the pulse tension is low. Collateral evidence in the shape of nocturnal rising, low specific gravity, headaches, slight transient œdema are helpful; and it may be held that cardiac dilatation in non-alcoholic patients, in the absence of primary valvular mischief, is of kidney origin. The diagnosis of mitral stenosis is difficult in the absence of murmur and thrill. Perhaps the most important physical sign is the sharp systolic rap or snap to be detected by palpation. This is usually best felt over the right ventricle, and is most marked before a tricuspid systolic bruit develops, but may be present afterwards. Another sign is systolic retraction in the interspace or spaces above the apex, which is very common in mitral stenosis, and is quite independent of pericardial adhesion. Increase in size of the right ventricle and auricle, with high-pitched second pulmonic sound, are very characteristic of the condition.

**Cardiac Disease and Pulmonary Tuberculosis.**—In a paper by Anders[2] the association of cardiac disease and pulmonary tuberculosis is discussed. True tubercular endocarditis is very rare, for the endocardium, like the tunica intima of an artery, is very resistent to the tubercle bacillus. It practically never occurs in the course of pulmonary tuberculosis. Almost all the rare instances of true tuberculosis of the endocardium are associated with other forms of cardiac tuberculosis, especially the pericarditic. A sclerotic form of endocarditis, which is due to tuberculous affection is sometimes met with, probably caused by an intense and protracted intoxication of the system, but no tuberculous changes are found in the thickened areas. Endocarditis may occur during the progress of phthisis due to streptococci or staphylococci, whilst acute rheumatism rarely complicates phthisis. The most important class of case is where a tuberculous pulmonary affection is superadded to a cardiac disease which has been caused by rheumatism. Valvular disease of the left side of the heart seems to confer, to some extent, immunity against pulmonary phthisis, possibly owing to the pulmonary congestion caused thereby; whilst stenosis of the pulmonary artery predisposes to phthisis, thus it is stated that 33 per cent. of such cases which survive the twentieth year die of phthisis.

**Myocardium.**—Gibson,[3] in an address on some affections of the myocardium, calls attention to the intimate connection of the myocardium with the vascular supply of the heart. It seems a little curious that the left coronary artery is smaller than the right; the explanation, however, lies in the fact that the right coronary artery supplies almost the entire right ventricle, the whole of the auricles, and the posterior aspect of the left ventricle, whilst the left one supplies little save the anterior wall of the left ventricle and a small portion of the adjacent anterior wall of the right ventricle. When there is a total sudden occlusion of one of the great coronary arteries there is invariably sudden death. There is no alteration in the tissue, it is simply anæmic, there having been no time allowed for any definite changes. Such an event, which is always due to embolism, is, however, very rare. A gradual blocking of one of the coronary arteries is much more often seen, due to sclerosis and subsequent thrombosis. This causes disappearance of the muscular fibres and their replacement by connective tissue, which is more or less fibrous according to the time the process has been going on. A heart which has undergone such alterations is liable to fail when there is any stress on it. This is the explanation why acute infections, and especially pneumonia, take on such a very serious aspect after middle life. It is difficult to lay down any definite rules by which a certain diagnosis can be made. The most important signs are symptoms of slight heart failure accompanied by evidence of degenerated arteries. Fatty degeneration produces much the same results, but apart from being a result of certain toxic processes, it only occurs as a part of general obesity. Hyaline degeneration is a result only of infections, such as diphtheria and pneumonia. Thus in elderly persons the diagnosis is often not so very difficult. In treatment no drug is of such importance as one or other of the iodides, together with general hæmatinics and tonics. The general environment and mode of life of the patient should be carefully attended to. Probably there is scarcely such a condition as a primary disease of the myocardium. The only primary affection which we can recognise is what may be called myocardial insufficiency. This is found in young people who suffer from cardiac failure after exertion which in a normal individual would be insufficient to cause it. The patient must be kept in bed until the heart has recovered its normal condition; and subsequently exercise must be carefully regulated.

[1] Brit. Med. Jour., Nov. 1. [2] Med. Chron., July. [3] Clin. Jour., June 11.

